# Rare variants in the GABA_A_ receptor subunit ε identified in patients with a wide spectrum of epileptic phenotypes

**DOI:** 10.1002/mgg3.1388

**Published:** 2020-06-25

**Authors:** Fenja Markus, Chloé Angelini, Aurelien Trimouille, Gabrielle Rudolf, Gaetan Lesca, Cyril Goizet, Eulalie Lasseaux, Benoit Arveiler, Marjon van Slegtenhorst, Alice S. Brooks, Rami Abou Jamra, Georg‐Christoph Korenke, John Neidhardt, Marta Owczarek‐Lipska

**Affiliations:** ^1^ Junior Research Group Genetics of Childhood Brain Malformations Faculty VI‐School of Medicine and Health Sciences University of Oldenburg Oldenburg Germany; ^2^ Human Genetics Faculty VI‐School of Medicine and Health Sciences University of Oldenburg Oldenburg Germany; ^3^ Service de Génétique médicale CHU de Bordeaux Bordeaux France; ^4^ CNRS U7104 INSERM U1258 Institut de Génétique et de Biologie Moléculaire et Cellulaire Illkirch France; ^5^ Service de Neurologie Centre de Références des Maladies Neurogénétique Rares Hôpitaux Universitaires de Strasbourg Strasbourg France; ^6^ Genetics department Lyon University Hospital and University of Lyon Lyon France; ^7^ Department of Clinical Genetics Erasmus MC University Medical Center Rotterdam The Netherlands; ^8^ Institute of Human Genetics University Medical Center Leipzig Leipzig Germany; ^9^ Department of Neuropediatrics University Children’s Hospital Oldenburg Germany; ^10^ Research Center Neurosensory Science University of Oldenburg Oldenburg Germany

**Keywords:** exome sequencing, GABA receptor type A subunit epsilon, GABRE, genetic epilepsy, novel sequence variants

## Abstract

**Background:**

Epilepsy belongs to a group of chronic and highly heterogeneous brain disorders. Many types of epilepsy and epileptic syndromes are caused by genetic factors.

The neural amino acid y‐aminobutyric acid (GABA) is a major inhibitory neurotransmitter in the mammalian central nervous system. It regulates activity of channel pores by binding to transmembrane GABA‐receptors (GABRs). The GABRs are heteropentamers assembled from different receptor subunits (α1‐6, β1‐3, γ1‐3, δ, ε, θ, π, and ρ1‐3). Several epileptic disorders are caused by mutations in genes encoding single GABRs.

**Methods:**

We applied trio‐ and single‐whole exome sequencing to search for genetic sequence variants associated with a wide range of epileptic phenotypes accompanied by intellectual disability and/or global developmental delay in the investigated patients.

**Results:**

We identified four hemizygous sequence variants in the GABA_A_ receptor subunit ε gene (*GABRE*), including one nonsense (NM_004961.3: c.399C>A, p.Tyr133*), two missense variants (NM_004961.3: c.664G>A, p.Glu222Lys; NM_004961.3: c.1045G>A, p.Val349Ile), and one variant affecting the translation initiation codon (NM_004961.3: c.1A>G, p.Met1?) in four unrelated families.

**Conclusion:**

Our clinical and molecular genetic findings suggest that *GABRE* is a likely candidate gene for epilepsy. Nevertheless, functional studies are necessary to better understand pathogenicity of the *GABRE*‐mutations and their associations with epileptic phenotypes.

## Graphical abstract

The neural amino acid y‐aminobutyric acid (GABA) regulates activity of channel pores by binding to transmembrane GABA‐receptors (GABRs). We identified four hemizygous sequence variants in the GABA_A_ receptor subunit ε gene (GABRE) in four epileptic patients. Our clinical and molecular genetic findings suggest that *GABRE* is a likely candidate gene for epilepsy.

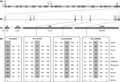

## 1 INTRODUCTION

The International League Against Epilepsy (ILAE) defined epilepsy as a prevalent and highly heterogeneous disorder of the brain (Fisher et al., 2014). This disorder leads to seizures, defining sudden erroneous and unprovoked electrical conductions between neurons (Guerreiro, 2016). The ILAE announced a three‐level classification system for epileptic seizure types, epilepsy types, and epileptic syndromes that support a better clinical diagnosis and treatment of patients affected with epilepsy (Chang, Leung, Ho, & Yung, 2017). Among epileptic seizure types three major groups can be distinguished: (a) focal onset seizures derived from neuronal networks which are exclusively restricted to an unilateral brain hemisphere, (b) generalized onset seizures which originate from bilateral neuronal networks, and (c) idiopathic epilepsies without any evident etiology, frequently showing genetic predispositions (Berg et al., 2018; Chang et al., 2017; Guerreiro, 2016; Hirose, Mitsudome, Okada, & Kaneko, 2005). Additionally, epileptic disorders were classified into genetic, structural, metabolic, and unknown origin (Berg et al., 2010; Korff & Scheffer, 2013).

Genetic factors play an important role in many types of epilepsy and epileptic syndromes, often in these with unknown origin (Nieh & Sherr, 2014). Despite of a fast development in bio‐medical technologies and advances in next generation sequencing (NGS), resulting in a substantial progress in the discovery of genes and/or multiple‐loci associated with epileptic disorders, many of the genotype‐phenotype correlations remain to be elucidated (Symonds, Zuberi, & Johnson, 2017). It has been previously reported that certain forms of epilepsy can be caused by pathogenic variants in several distinct gamma‐aminobutyric acid receptor (GABR) subunit genes (Butler et al., 2018; Hernandez et al., 2016b; Ishii et al., 2017; Shen et al., 2017). GABA is the major inhibitory neurotransmitter in the mammalian central nervous system (CNS), involved in numerous developmental processes in the brain (Macdonald & Olsen, 1994; Watanabe, Maemura, Kanbara, Tamayama, & Hayasaki, 2002). It is involved in neural migration, facilitates extension of neurites, and plays an important role in a synaptic maintenance (Behar et al., 1994; Behar, Schaffner, Scott, O'Connell, & Barker, 1998; Watanabe et al., 2002). About 20% of neurons in the CNS are GABAergic (Watanabe et al., 2002). GABRs are pentameric, ligand‐gated chloride channels, composed of a combination of different subunits (α1‐6, β1‐3, γ1‐3, δ, ε, θ, π, and ρ1‐3; Hernandez et al., 2016b; Macdonald & Olsen, 1994; Watanabe et al., 2002). Each GABR subunit is composed of a large extracellular N‐terminal domain, four hydrophobic transmembrane domains, and a large intracellular domain, which is the most heterogeneous part of a single receptor (Moss & Smart, 2001). Ionotropic GABRs are classified based on their pharmacology into two types of receptors: GABA_A_ and GABA_C_ (Moss & Smart, 2001). GABA_A_ receptors are important targets of drugs, including benzodiazepines, barbiturates, and other neuroactive medications (Sieghart, 2000). Pathogenic variants in genes encoding for several GABA_A_ receptor subunits are known to cause epilepsies of different severity, from mild febrile seizures to severe epileptic encephalopathies (Carvill et al., 2014; Cossette et al., 2002; Hernandez et al., 2016b; Kodera et al., 2016; Moller et al., 2017; Shen et al., 2017). The majority of monogenic pathogenic variants are associated with *GABRA1*, *GABRB2/3*, and *GABRG2* genes, which form the abundant α1β2/3γ2 subtype of GABA_A_ receptors in the CNS (Farrant & Nusser, 2005). Unlike many GABA_A_ receptors, those containing the ε subunit have restricted distributions, form functional channels with distinct pharmaco‐biochemical activities and lead to spontaneous channel opening with slowed recovery (Neelands, Fisher, Bianchi, & Macdonald, 1999; Wagner, Goldschen‐Ohm, Hales, & Jones, 2005). The assembly of the ε subunit into GABA_A_ receptors and its complete biological function is poorly understood (Bollan, Baur, Hales, Sigel, & Connolly, 2008). So far, only a few variants of unknown significance in the GABA_A_ receptor subunit ε gene (*GABRE*, OMIM: 300093) have been reported in single epilepsy‐cases (Butler et al., 2018; Hernandez et al., 2016b; Wang, Du, et al., 2017).

Herein, we present four novel hemizygous sequence variants identified in the *GABRE* gene, including nonsense (*n* = 1), missense (*n* = 2), and translation initiation codon (*n* = 1) variants, in unrelated patients manifesting various epileptic phenotypes. Our clinical and genetic findings extend the current set of known genes related to genetic epilepsies (GEs) and neurodevelopmental disorders. This will lead to a better understanding of pathogenic mechanisms underlying these devastating neurological, frequently life‐threatening conditions, and may also contribute to the development of improved antiepileptic drug‐based therapies in the future.

## 2 MATERIAL AND METHODS

### 2.1 Clinical data of patients and ethical compliance

GE affected patients (P1, P2, P3, and P4) and their available family members were referred to the Human Genetics at the University of Oldenburg (Oldenburg, Germany), CHU de Bordeaux, Hospices Civils de Lyon (Lyon, France), and Erasmus MC University Medical Center (Rotterdam, the Netherlands). All examined relatives of P1 originated from Germany, whereas P2, P4, and their relatives originated from France. The family of P3 settled in the Netherlands, but the grandparents of this patient were of Russian Caucasian origin. The patients and their relatives were informed about possible consequences of the study, agreed to participate in the study, and signed informed written consents before the project started. The study adhered to the tenets of the Declaration of Helsinki and was approved by local ethics committees (Hannover Medical School (MHH) ethics committee, OE9515; Medizinische Ethik‐Kommission University Oldenburg, Comité de protection des personnes Bordeaux‐ Outre Mer III, France; Erasmus MC, METC‐2012387).

### 2.2 DNA extraction

Peripheral blood samples from the patients and their available family members were collected in EDTA tubes. DNA isolations were performed according to the manufacturer's recommendations using either Gentra Puregene Kit (QIAGEN) or Nucleon BACC3 (GE Healthcare) or Tecan EVO workstation (Promega). DNA quantity and quality were measured using, either Biospectrometer (Eppendorf) or Qubit^®^ Fluorometer (Thermo Fisher Scientific) or NanoDrop spectrophotometer (Thermo Fisher Scientific).

### 2.3 Whole exome sequencing (WES)

A total of 50 ng of genomic DNA (gDNA) from P1 was used for an enzymatic fragmentation/ligation and enrichment of exomic sequences using TruSeq^®^ Rapid Exome Kit (Illumina). DNA libraries obtained from gDNA of P1 were 3‐plexed with two different DNA libraries that were not included in this study. A pair‐end sequencing (2 × 75 bp) using NextSeq™ 500 Mid Output Kit was conducted with the NextSeq500 system (Illumina). Demultiplexing and processing of WES data, together with variant calling, and annotation were performed using Varvis™ bioinformatics platform (Limbus Medical Technologies GmbH) based on the human genome build (hg19/GRCh37). High and moderate impact genetic sequence variants were filtered for allele frequency (AF) below 1.5% reported in Exome Aggregation Consortium (ExAc, https://gnomad.broadinstitute.org/) and Genome Aggregation Database (GnomAD, https://gnomad.broadinstitute.org/). The sequence variants following X‐linked and autosomal recessive modes of inheritance were considered for further analyses. Sequencing reads encompassing selected sequence variants were additionally evaluated with Integrated Genomics Viewer (IGV, http://software.broadinstitute.org/software/igv).

Trio‐WES analysis was performed for family 2, including P2 and unaffected parents. Preparation of gDNA libraries, exome capture, sequencing, and data analysis were made with IntegraGen SA (IntegraGen SA), using SureSelect XT Clinical Research Exome in‐solution enrichment methodology (Agilent technologies, Santa Clara, California, USA). Paired‐end sequencing runs (2 × 75 bp) were performed on Illumina HiSeq4000 (Illumina). The detailed explanations of the process have been previously described (Gnirke et al., 2009). A bioinformatic pipeline, including base calling was performed for each WES data using the Real‐Time Analysis software version 2.7.7 (Illumina) sequence pipeline with default parameters. Sequence reads were mapped to the hg19/GRCh37 using Elandv2e (CASAVA1.8.2; Illumina), and duplicated reads were removed. Furthermore, CASAVA1.8.2 was used to call single‐nucleotide variants (SNVs) and short insertions/deletions (max. size is 300 bp). Variants’ filtering was performed on Eris v3.0 (IntegraGen SA). Only variants with an appropriate familial segregation were considered, under the hypothesis of an autosomal recessive, X‐linked and a dominant disease with de novo germinal pathogenic variants. Variants with minor allele frequency (MAF) ≥1.5% in ExAc as well as noncoding variants were not included into further analyses. Finally, mapped reads were visualized with Alamut Visual Software 2.9.0 (Interactive Biosoftware).

Trio‐WES was applied in family 3 using the Agilent Sure Select platform (Clinical Research Exome V2). A 150 bp paired‐end sequencing run was performed on HiSeq4000 (Illumina). Sequencing data were demultiplexed by CASAVA software (Illumina) and reads were mapped to the hg19/GRCh37 by Burrows‐Wheeler Aligner (http://bio‐bwa.sourceforge.net/). Variants were detected with Genome Analysis Toolkit (http://www.broadinstitute.org/gatk/). Subsequently, sequence variants were filtered with Cartagenia software package (Agilent Technologies), considering reads’ quality (read depth ≥10), MAF ≤ 1.5% (dbSNP, ESP6500, the 1,000 Genome project, GoNL, or the ExAc database), and location (within an exon and 10 bp areas encompassing introns). Variants were further selected in regard to an autosomal recessive, X‐linked or de novo dominant inheritance.

Trio‐WES analyses of family 4 using DNA samples from P4 and healthy parents were performed on a HiSeq 1000 Sequencing System (Illumina). The sequencing was done with a 2 × 150 bp high output sequencing kit after enrichment with SeqCap EZ MedExome kit (Roche), according to manufacturer's specifications. Sequence quality was assessed with FastQC 0.11.5 (Babraham Bioinformatics). After sequencing, reads were mapped to the hg19/GRCh37, using BWA‐MEM (http://bio‐bwa.sourceforge.net), sorted and indexed (Samtools, http://samtools.sourceforge.net). Duplicates were flagged (Sambamba, https://www.open‐bio.org/wiki/Sambamba) and coverage was calculated (Picard‐Tools, https://broadinstitute.github.io/picard/). Variant calling was performed with GATK 3.7 Haplotype Caller (https://gatk.broadinstitute.org/hc/en‐us) and variants were annotated with SnpEff (http://snpeff.sourceforge.net/), dbNSFP (http://sites.google.com/site/jpopgen/dbNSFP), GnomAD, ClinVar (https://www.ncbi.nlm.nih.gov/clinvar/), Human Gene Mutation Database (HGMD), Variome Great Middle East (http://igm.ucsd.edu/gme/data‐browser.php) Mutation Tester (http://www.mutationtaster.org/), Combined Annotation Dependent Depletion (CADD, https://cadd.gs.washington.edu/), as well as with an in‐house‐database.

### 2.4 Primers, PCR amplification and Sanger sequencing

**Figure 1:**
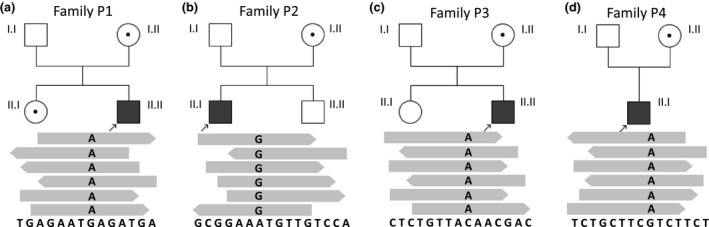
Analysis of identified hemizygous variants in *GABRE* in unrelated patients. (A) Family P1 includes healthy parents (I.I, I.II), one female healthy child (II.I), and the GE‐affected child (P1, II.II). The father (I.I) showed the hemizygous reference sequence, while the mother (I.II) and the sister (II.I) were carriers of the missense variant NM_004961.3: c.664G>A. (B) Family P2 consists of healthy parents (father I.I, mother I.II), one male healthy child (II.II) and one affected child (P2, II.I, NM_004961.3: c.1A>G). The other members of this family are healthy. (C) Family P3 includes the healthy parents (father I.I, mother I.II) and two children, the healthy daughter (II.I) and P3 (II.II), who showed a C‐to‐A substitution, leading to a premature termination codon (NM_004961.3: c.399C>A). The other members of family P3 were healthy. (D) Family P4 consists of healthy parents (father I.II, mother I.II) and one affected child (P4, II.I, NM_004961.3: c.1045G>A). No relevant familial history was noted. All variants were detected using WES and reads were obtained from both directions, forward and reverse. Symbols: squares = males; circles = females; black solid squares with arrows = patients affected with epilepsy; solid white icons = healthy individuals; icons with dot = known carrier

Sanger sequencing was performed in family 1. Primers (fwd_5’‐TAAGAGAGGCAAGGTCCCAC, rev_5’‐CTTGGCAAAGCACTTACTTCTT) located in intronic regions of the human *GABRE* gene (NM_004961.3) and encompassing exon 6 were designed using Primer Input3 (http://primer3.ut.ee/). They were additionally verified for common SNPs using SNPCheck (https://secure.ngrl.org.uk/SNPCheck/). In total, 10 ng of gDNA samples from P1 and from unaffected relatives (including parents, sister, and grandfather from maternal side) were used for PCR amplification with HotFirePol DNA Polymerase (Solis BioDyne), according to manufacturer's recommendations (Figure 1a; Figure S1a). Afterward, the amplicons were purified using enzymatical ExoI‐SAP method (New England Biolabs) and bilaterally sequenced using the BigDye^®^ Terminator v3.1 Cycle Sequencing Kit on the ABI Prism 3130xl Genetic Analyzer (Applied Biosystem). Sanger sequencing data were analyzed with SeqScape software (Applied Biosystem) and SnapGene software (GSL Biotech).

Results of high throughput sequencing from P2, P3, and P4 were not additionally verified by Sanger sequencing as the *GABRE* gene, especially the nucleotide positions of the identified pathogenic sequence variants were very well covered in the trio‐WES data.

### 2.5 Multiple species protein alignments and in silico predictions of GABRE protein domains

Multiple sequence alignments were performed with the ClustalW2 tool (http://www.ebi.ac.uk/Tools/msa/clustalw2/). Amino acid sequences of GABRE proteins were compared between human (GenBank: NP_004952.2), mouse (GenBank: NP_059065.2), Norway rat (GenBank: NP_075579.1), rabbit (GenBank: XP_002721372.1), hedgehog tenrec (GenBank: XP_004715681.1), Sunda flying lemur (GenBank: XP_008589892.1), dog (GenBank: XP_549340.3), Californian Sea Lion (GenBank: XP_027465429.1), Chinese rufous horseshoe bat (GenBank: XP_019566807.1), and lesser ferret (GenBank: XP_004776709.1).

Publicly available tools to predict protein domains, such as NCBI Conserved Domain Search (https://www.ncbi.nlm.nih.gov/Structure/cdd/wrpsb.cgi), PROSITE (http://prosite.expasy.org), and SMART (http://smart.embl‐heidelberg.de) were used to in silico estimate locations of the mutated amino acids in the GABRE protein, found in our patients.

Additionally, MetaDome data (https://github.com/cmbi/metadome) were used to predict pathogenicity and improve interpretation of the newly identified GABRE‐variants. MetaDome aligns information of population variations from the ExAc and pathogenic variants from the HGMD with Pfam protein domains. Generally, MetaDome visualizes meta‐domain information and gene‐wide profiles of genetic tolerance based on 56’319 human transcripts, 71’419 protein domains, 12’164’292 genetic variants from GnomAD, and 34’076 pathogenic variants from ClinVar (Wiel et al., 2019).

## 3 RESULTS

### 3.1 Clinical features of epilepsy‐affected patients

P1 is a 5 ½ years old boy and the only affected child of non‐consanguineous parents (Figure S1a). Pregnancy and birth at term were normal (birth weight 3,905 g, body lengths 53 cm, head circumference 39.0 cm). Apgar values were normal with 9‐10‐10. Myoclonic jerks were observed at the age of 4 weeks. The first electroencephalography (EEG) was performed at the age of 8 weeks and showed no epileptic discharges. At the age of 3 months P1 was transferred to University Children's Hospital, Klinikum Oldenburg, Oldenburg because of increased infantile spasms. Because EEG showed hypsarrhythmia the West syndrome was diagnosed in P1. Extensive diagnostic investigations, including cerebral magnetic resonance imaging (MRI), metabolic examinations with cerebrospinal fluid examination with neurotransmitter analysis, genetic array comparative genomic hybridization (array CGH), and panel diagnostic for epileptic encephalopathies (79 genes) were performed. However, no abnormalities were detected. An extended antiepileptic therapy over the next years with 11 different antiepileptic drugs and a ketogenic diet were applied. However, these treatments did not give any long‐lasting positive therapeutic effects and the patient was finally diagnosed with pharmaco‐resistant epilepsy of the West syndrome type (Table 1, P1). Recently, (at the age of 5 ½ years) P1 was treated with valproic acid, sulthiame, and cannabidiol. Renew EEG examinations showed a too slow basic rhythm, bioccipital delta waves, multifocal sharp‐ and sharp‐slow‐waves predominantly from temporo‐parietal. Furthermore, P1 showed a strong abnormal psychomotor development. At the age of 5½ years P1 was unable to fix or follow objects with his eyes, he was unable to grasp, to turn around or to sit freely. He was not able to spell out and understand any words. The head circumference declined over the time and the patient developed a microcephaly (48 cm, first percentile, −2.9 *SD*). MRI examinations were additionally performed at the age of 4 and 7 months, and 3 and 4 years. MRI findings revealed a progressive global cerebral atrophy.

**Table 1:** Clinical and genetic features of the investigated patients

Patient ID	Gender/age of onset	Likely pathogenic variant	Diagnosis	Development	Seizure type	Medication	EEG	MRI
P1	M/2 m	c.664G>A p.Glu222Lys	Severe pharmaco‐resistant epileptic encephalopathy, West syndrome	Abnormal psychomotor development, severe global developmental delay and intellectual disability, microcephaly	Initial myoclonic seizures, 3 m: infantile spasms	Pyridoxalphosphate, pulsatile dexamethason, prednisolone, vigabatrin, topiramate, valproic acid, levetiracetam, ketogenic diet, oxcarbazepin (aggravation), lacosamide actual: valproic acid, sulthiame, cannabidiol	Hypsarrhythmia, slow basic rhythm, bioccipital delta waves, multifocal sharp‐ and sharp‐slow‐waves	Abnormal: progressive global cerebral atrophy
P2	M/6 y	c.1A>G p.Met1?	Focal epilepsy	Global developmental delay, mild static and kinetic ataxia, intellectual disability	Focal seizures	Valproic acid, levetiracetam, topiramate, sulthiame	Temporal abnormalities	Normal
P3	M/18 y	c.399C>A p.Tyr133*	Generalized epilepsy	Autistic behaviors, dysmorphic signs, intellectual disability	Tonic‐clonic seizure	Valproic acid, levetiracetam, carbamazepine, risperidone, methylphenidate	Epileptiform abnormalities fronto‐polar on both sides and left occipital with low index	NORMAL
P4	M/3 y	c.1045G>A p.Val349Ile	Generalized epilepsy	Global developmental delay, intellectual disability	Focal to bilateral seizures	Ospolot, urbanyl, sulthiame, clobazam	Spikes in contralateral hemisphere, bilateral wave discharges	normal
a_2_ (Wang, Du, et al., 2017)	M/6 m	c.1355G>T p.Arg452Leu	Infantile spasms	Severe global developmental delay and intellectual disability	Spasms in cluster, infantile spasms	Valproic acid, topiramate, clonazepam	Hypsarrhythmia, multiple spike, and slow wave complex	Myelin development delay, brain dysplasia

Note

Patient a_2_ described by Wang, Du, et al. (2017). Sequence variants were annotated using reference sequence NM_004961.3/NP_004952.2.

Abbreviations: EEG, electroencephalography; M, male; m, month; y, year.

P2 is 21 years old and the only affected child in a non‐consanguineous family. There is a familial history of intellectual deficiency in the maternal branch, with multiple male affected with intellectual disability (Figure S1b). P2 was born at term after an uneventful pregnancy. He presented global developmental delay, was able to sit alone at the age of 10 months and walked at the age of 30 months. Mild static and kinetic ataxia were observed since the age of 3 years, and remained stable over years. He presented partial complex epileptic seizures with temporal abnormalities on EEG at the age of 6 years (Table 1). He was effectively treated with valproic acid, levetiracetam, and topiramate, showing normal EEGs at the age of 10 years. He also had a bicuspid aortic valve and small initial aorta dilatation, treated with bisoprolol. Dilatation remained stable over years. Hyperprolinemia was noted during an amino acid chromatography testing, which was treated with riboflavin. He followed adapted courses during childhood and was finally referred to a center for disabled persons.

P3 is a 21‐year‐old man and one of two children of unrelated parents (Figure S1c). He was born at a gestational age of 42 weeks with a birth weight of 3,850 g. P3 pronounced his first words at the age of 1.5 years, in a context of bilingualism (Dutch and Russian). Initial concerns appeared around the age of 2 years, with hyperactive behavior at the age of 3 years. He also manifested speech delay, and autistic behaviors. Intelligence quotient (IQ) testing revealed an IQ score of 55. At the age of 18 years he had a first tonic‐clonic seizure, without clearly provoking factors. He was treated with valproic acid, levetiracetam, and carbamazepine. A valproic acid medication alone was insufficient to control his epilepsy (Table 1). To improve attention abilities and better control behavioral disturbances the patient was additionally treated at the age of 8 years with risperidone and methylphenidate. He did not show any visual or auditive impairment. Physical examination identified dysmorphic signs, similar to Marfanoid habitus, with narrow and high palate, long and slender fingers with slight camptodactyly, mild pectus excavatum, and stretch marks on the chest, shoulder, and lower back.

P4 was 10 years old at the time of the study. He was born after an uneventful pregnancy (Figure S1d). He presented global developmental delay during childhood and manifested behavior troubles. He suffered from focal to bilateral seizures since the age of 3 years (Table 1). EEG‐measurements showed highlighted spikes in contralateral hemisphere as well as bilateral wave discharges. He was treated with sulthiame and clobazam. Brain MRI examination did not detect any abnormalities. He also presented hypothyroidism.

### 3.2 Sequencing analyses

**Figure 2:**
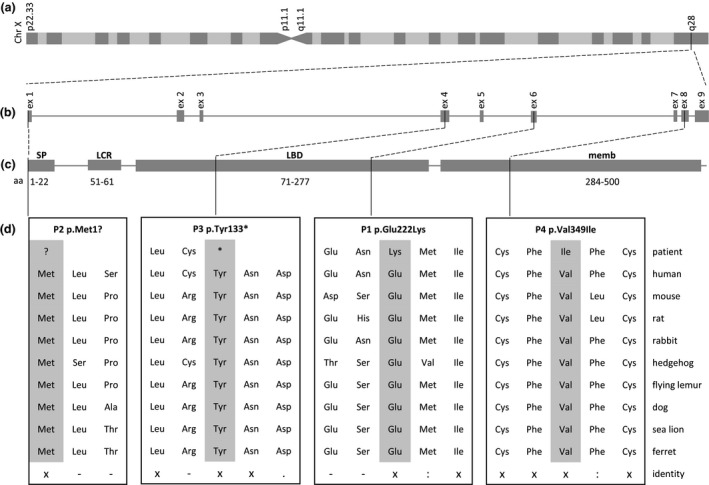
Identification of sequence variations in the GABRE gene in 4 GE‐affected patients. (A and B) X‐chromosomal localization of the human *GABRE* gene and its distribution of exons. (C) We found variants in exon 1, 4, 6, and 8, located in different protein domains. (D) Multiple species sequence alignments of GABRE proteins. The mutated GABRE protein sequences of the patients described herein were shown on top of the alignments. Consensus symbols in the alignments represent: aa, amino acid; SP, signal peptide; LCR, low complexity region; LBD, Pfam Neurotransmitter‐gated ion‐channel ligand binding domain; memb, Pfam Neurotransmitter‐gated ion‐channel transmembrane region; (x), single; fully conserved residues; (:), strongly similar residues; (.), weakly similar residues; (−), lack of identity between residues

The DNA library from P1 was multiplexed with two different DNA libraries, which were not subjected to the project. The 3‐plex sequencing run resulted in 31.8 Gb raw data and showed a Q30‐score of 87%, cluster density of 336 K/mm^2^, and cluster passing filter of 74.9%. Size of sequencing data obtained from P1 was estimated to 6.70 Gb, generating 66.86 M of clusters, 133.72 M of reads, and 92.5% of 10× targeted coverage. Applying stringent selection settings, the initial number of genetic variants was reduced to 90. We verified these variants for plausible types of inheritance, including autosomal‐recessive and X‐linked. Only one genetic variant passed the filtering criteria of X‐linked mode of inheritance. The remaining 89 variants were excluded either due to an incorrect inheritance pattern or misguided disease‐associations. We also considered heterozygous variants for possibly being de novo variants. None of these heterozygous variants was found in a promising candidate gene, to further verify its potential de novo appearance in familiar co‐segregation analyses with Sanger sequencing (Table S2). According to the hg19, this genetic variant was located on X‐chromosome, at position 151’128’431. It was a missense variant (NM_004961.3: c.664G>A, NP_004952.2: p.Glu222Lys) located in exon 6 of the *GABRE* gene (Figures 1 and 2). Out of a total of 28 aligned reads, 22 were sequenced from a reverse direction, whereas 6 reads were shown from a forward direction. All sequenced reads contained the G‐to‐A exchange at the nucleotide position 664 (Figure 1a). The allele frequency of the c.664G>A (NM_004961.3) substitution, was estimated to 0.00004409 ExAc (Table S1). In silico predictions of the glutamic acid into lysine substitution at the amino acid position 222 in the GABRE protein suggested a likely pathogenic variant and showed M‐CAP = 0.387 and SIFT = 0.78 (VARVIS Version 1.12; Limbus Medical Technologies GmbH). Sanger sequencing was additionally performed in P1 and the available family members. The analyses confirmed the co‐segregation of the c.664G>A (NM_004961.3) variant in the *GABRE* gene in the family of P1 (Figure 1a; Figure S1a).

In family 2, a trio‐WES was performed. The sequenced data for P2 resulted in 9.69 Gb raw data and showed Q30‐score of 97%, and 95% of bases were covered at 25×. After variant filtering, only one variant was identified as a potential candidate for GE (Table S2). This variant was located on the X‐chromosome at position 151’974’625. The sequence variant was detected in exon 1 of the *GABRE* gene and was thought to affect the translation initiation codon (NM_004961.3: c.1A>G, NP_004952.2: p.Met1?). Out of 63 aligned reads, 42 were sequenced from a reverse direction, whereas 21 reads were shown from a forward direction. The allele frequency of this sequence variant was estimated to 0.00001550 (ExAc, Table S1). The single A‐to‐G substitution at nucleotide 1 of the *GABRE* gene in P2 was predicted to likely cause an alternative initiation codon. This substitution was found in all sequencing reads. The translation initiation codon ATG (coding for methionine) was changed to GTG (coding for valine). Consequently, this variant was predicted to result in an abnormal length of the GABRE protein (NP_004952.2: p.Met1?). This variant co‐segregated with the GE phenotype within the family (Figure 1b).

A gene‐panel approach for intellectual disabilities that was initially applied in P3 did not reveal any obvious causative variants. Therefore, trio‐WES was considered to further search for the molecular cause of GE in P3. Around 95% of sequencing reads from each WES showed a coverage of 10×. Simultaneous analyses of WES data for P3 and his healthy parent revealed a nonsense variant in the *GABRE* gene (NM_004961.3: c.399C>A, NP_004952.2: p.Tyr133*). P3 was hemizygous for the mutated allele, whereas his mother was a carrier, and the father showed the reference allele (Figure 1c).

Variant filtering was applied in P4 and his healthy parents. A hemizygous missense variant, located in exon 8 of the *GABRE* gene was only found in P4, whereas the mother was carrier for this variant. As expected, the unaffected father showed reference allele (Figure 1d). The identified *GABRE*‐variant in P4 was predicted to lead to an exchange of valine into isoleucine (NM_004961.3: c.1045G>A, NP_004952.2: p.Val349Ile) and was not found in the ExAc/GnomAD database. SIFT score and Mutation Tester showed scores of 0.92 and 0.947, respectively (Table S1). The score of the CADD was estimated to 6.512. These predictions support the pathogenicity of the variant in the *GABRE* gene detected in P4. In total, 20 x coverage of each WES was obtained for about 93% of sequencing reads.

### 3.3 Bioinformatic predictions of the identified variants in the GABRE protein

To evaluate the evolutionary conservation of the GABRE protein, multiple protein alignments of different species were performed and showed a full conservation at amino acid 1, 133, 222, and 349 in regard to the human reference protein (NP_004952.2; P2, P3, P1, P4; Figure 2).

**Figure 3:**
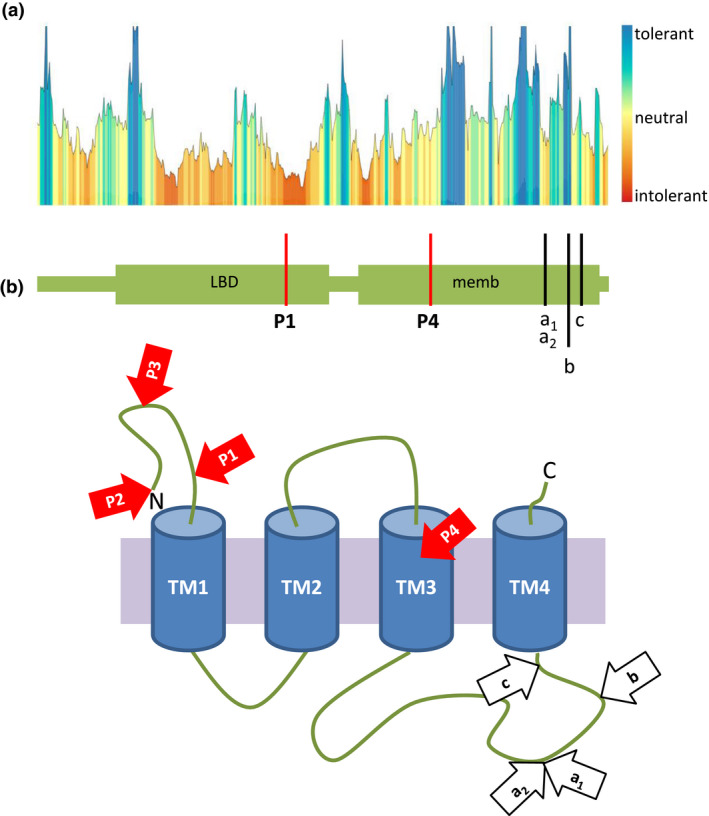
Schematic presentation of pathogenicity predictions and localization of variants in the GABRE protein. (A) Landscape of the localization of the novel and previously identified variants in the *GABRE* gene and their predicted tolerance in the specific regions of the GABRE protein performed with MetaDome (NM_004961.3, NP_004952.2). Red areas represented highly intolerant regions, whereas blue regions showed highly tolerant areas. (B) The newly identified GABRE‐variants are presented as red arrows (P2, NP_004952.2: p.Met1?; P3, NP_004952.2: p.Tyr133*; P1, NP_004952.2: p.Glu222Lys, intolerant; P4, NP_004952.2: p.Val349Ile, slightly intolerant). The previous GABRE‐variants reported by Hernandez et al., 2016 (a_1_: p.Arg452Gly, intolerant; b: p.Arg472His, tolerant, and c: p.Ser484Leu, neutral) and by Wang, Du, et al., 2017 (a_2_: p.Arg452Leu, intolerant) are shown as white arrows. TM, transmembrane region (TM1: aa 254‐274; TM2: aa 281‐301; TM3: aa 344‐364; TM4 aa 486‐506); aa, amino acid; N, N‐terminus; C, C‐terminus; LBD, Pfam Neurotransmitter‐gated ion‐channel ligand binding domain; memb, Pfam Neurotransmitter‐gated ion‐channel transmembrane region.

In silico predictions of the GABRE protein domains revealed that the genetic variants identified in P1 (NP_004952.2: p.Glu222Lys) and P3 (NP_004952.2: p.Tyr133*) were both located in the neurotransmitter‐gated ion‐channel ligand binding domain (PF02931; Figures 2c and 3b). This protein domain plays an important role in ligand binding, which in turn leads to changes in protein conformation and channel opening (Richter et al., 2012). The inflow of chloride into the cell is thought to result an inhibitory postsynaptic potential. The variant identified in P2 (NP_004952.2: p.Met1?) was located at the signal peptide site of the GABRE protein, which determines the localization and the transport pathway of the protein within the cell (Blobel, 1980). The missense variant found in P4 (NP_004952.2: p.Val349Ile) was mapped to the neurotransmitter‐gated ion‐channel transmembrane region domain (PF02932). This protein domain is essential for ion channel formation (Figures 2c and 3b; Chen et al., 2018).

Tolerance analyses of the missense variants described herein (NM_004961.3: P1 c.664G>A, NM_004961.3: P4 c.1045G>A) revealed that the mutated amino acids were located in functional regions of the GABRE protein with intolerant and slightly intolerant impacts (Figure 3a). Moreover, we also tested the impact of previously reported missense variants (NP_004952.2: p.Arg452Leu, NP_004952.2: p.Arg452Gly, and NP_004952.2: p.Arg472His) in GABRE (Hernandez et al., 2016a; Wang, Du, et al., 2017). The two variants located at arginine 452 were predicted to be intolerant, while substitutions at arginine 472 and at serine 484 were predicted to be highly tolerant or neutral, respectively (Figure 3b). These computational analyses of variants allow us to better understand the possible pathogenicity of the GABRE‐variants.

## 4 DISCUSSION

Hereditary epilepsies belong to a prevalent and highly heterogeneous group of brain disorders (Fisher et al., 2014). They lead to sudden, erroneous and unprovoked electrical currents in neuronal networks and are frequently caused by pathogenic variants in many different genes (Guerreiro, 2016; Wang, Lin, et al., 2017).

In this study, we identified four families with four novel sequence variants in the *GABRE* gene, including one nonsense (P3: NM_004961.3: c.399C>A, NP_004952.2: p.Tyr133*), two missense variants (P1: NM_004961.3: c.664G>A, NP_004952.2: p.Glu222Lys; P4: NM_004961.3: c.1045G>A, NP_004952.2: p.Val349Ile), and one affecting a translational initiation codon (P2: NM_004961.3: c.1A>G, NP_004952.2: p.Met1?) in unrelated patients with GEs.

The GABAergic signaling is a major biological pathway and its abnormal regulation in neurons is known to be associated with numerous CNS disorders (Kang, 2017; Kumar, Sharma, Kumar, & Deshmukh, 2013). Pathogenic variants in GABA_A_ receptor subunit genes, including *GABRA*, *GABRB*, and *GABRG*, impair GABAergic signaling via postsynaptic mechanisms that lead to GEs (Butler et al., 2018; Kang, 2017). Electrophysiological and biochemical studies suggested that the majority of pentamer receptors are composed of two α subunits, two β subunits, and a single γ subunit (Y. Chang, Wang, Barot, & Weiss, 1996; Tretter, Ehya, Fuchs, & Sieghart, 1997). This assembly of GABA_A_ subunits is frequently found in a human brain (Bollan et al., 2008). The ε subunit seems to be evolutionary related to the γ subunit, showing an identity of amino acid restudies above 40% (Davies, Hanna, Hales, & Kirkness, 1997). It has been speculated that the ε subunit is capable of replacing the γ subunit in the GABR pentamer, in order to form functional channels with distinct pharmacological and biophysical properties (Davies, Kirkness, & Hales, 2001; Saxena & Macdonald, 1994). This notion is supported by the finding that a replacement of α1 at the first position or β2 at the fourth position in the subunits’ assembly also resulted in functional channel pores (Bollan et al., 2008). Previous studies have further shown that pathogenic variants in α, β, and γ subunits significantly impair structures and functions of GABA_A_ receptors and lead to GEs (Hernandez et al., 2016b; Janve, Hernandez, Verdier, Hu, & Macdonald, 2016; Wang, Du, et al., 2017). The reported deleterious sequence variants have been mainly identified in N‐terminal regions and transmembrane protein domains, emphasizing an important biological function of these protein parts (Hernandez et al., 2016b). Interestingly, the *GABRE* sequence variants reported herein were also predicted to alter these protein regions supporting their involvement in GE.

Interactions of the ε subunit with α, β, and γ subunits are poorly understood and raise the question of their potential to form specific or unique neurotransmitter binding sites. To date, the impact of sequence variants located within the neurotransmitter‐gated ion‐channel ligand binding domain of ε subunits on a loss or gain of binding sites remains unclear. Unlike other GABR subtypes, the receptors containing a single ε subunit were described to act agonist‐independent and to spontaneously lead to slower deactivation and recovery of the receptors (Davies et al., 2001; Maksay, Thompson, & Wafford, 2003; Neelands et al., 1999; Ranna, Sinkkonen, Moykkynen, Uusi‐Oukari, & Korpi, 2006; Wagner et al., 2005). Pathogenic variants in a single α, β, and γ subunit of GABA_A_ receptors showed negative effects on opening and closing of channel pores changing their structure and/or conductive functions, and therefore, are thought to result in epileptic phenotypes (Matsumoto & Ajmonemarsan, 1964; Prince & Wilder, 1967). Since pathogenic variants in the α1 subunit lead to GEs (Burgess et al., 2019; Hernandez et al., 2016b, 2019; Krenn et al., 2019), it is likely that an exchange of α1 with a mutated ε subunit will cause epileptic phenotypes.

It has been shown that various pathogenic variants in the same GE‐associated gene may result either in different forms of neurodevelopmental disorders or cause a wide spectrum of epilepsies (Johannesen et al., 2016). In the patients described herein, we observed that sequence variants in the *GABRE* gene may cause a broad range of epileptic phenotypes, from an early‐onset and severe pharmaco‐resistant epileptic encephalopathy to late‐onset and mild forms of generalized and focal epilepsy (Table 1, Figure 3). Currently, only handful patients with *GABRE*‐variants were described worldwide and only very little details were published about the clinical symptoms of these patients. So far, only one patients with a missense variant (NM_004961.3: c.1355G>A, NP_004952.2: p.Arg452Leu) in the *GABRE* gene was described to manifest infantile spasms, hypsarrhythmia, myelin development abnormalities, and brain dysplasia (Wang, Du, et al., 2017). We also found similar clinical symptoms in P1 affected with the West syndrome. Interestingly, most of the patients presented herein showed moderate forms of epilepsy with developmental delay and intellectual disability. These are novel clinical features that were unreported in patients having sequence variants in the *GABRE* gene (Hernandez et al., 2016b; Wang, Du, et al., 2017). Interestingly, a wide spectrum of epileptic phenotypes, ranging from early‐onset severe forms, to late‐onset mild forms, and to cases with intellectual disability and developmental delay with other neuropsychiatric symptoms were already reported in patients with mutations in other GABA_A_ receptor subunit genes (Maljevic et al., 2019; Oyrer et al., 2018). However, due to a limited number of cases with sequence variants in the *GABRE* gene it is currently difficult to establish a clear phenotype‐genotype correlation. Moreover, modifier variants located in uncovered coding and noncoding regions of known epilepsy‐related genes, as well as variants in new genes, which cannot be excluded, may also impact on the phenotypic discrepancies observed in the investigated patients.

The available clinical data also suggested that the patients may respond differently to treatments with antiepileptic drugs. Epileptic seizures in P3 and P4 were either under control or even fully silenced in P2 due to applied therapies. However, seizures were not treatable or diminished by medications in P1, who showed pharmaco‐resistant epilepsy. It has been reported that many GE‐affected patients, similar to P1, did not respond to any drug therapies (Pierzchala, 2010). Consequently, epilepsies and epileptic syndromes remain life‐threatening neurological disorders. An identification of new pathogenic variants in epilepsy‐associated genes, and a discovery of novel candidate genes associated with epilepsies and neurodevelopmental disorders are essential for a deeper understanding of the genetic heterogeneity underlying these clinical symptoms. Additionally, in vitro and in vivo functional studies will be essential to enable the understanding of the molecular functions and pathogenicity of identified sequence variants, and thus, will contribute to a development of treatment options.

In this study, we identified novel sequence variants in the *GABRE* gene in four unrelated patients affected with GEs. The previously described genomic studies on large cohorts of patients affected with different forms of GE independently described four single cases with sequence variants in the *GABRE* gene (Hernandez et al., 2016b; Wang, Du, et al., 2017). However, the interpretation of these genetic findings was difficult due to the lack of comparable cases. Private pathogenic variants or sequence variants of unknown significance could not be excluded. Our genetic and clinical findings observed in P1 were similar to the previously reported case with *GABRE*‐variant (Wang, Du, et al., 2017) emphasizing an association of sequence variants in the *GABRE* gene with GE. We also collected three patients with *GABRE*‐variants showing milder epileptic phenotypes as well as developmental delay and intellectual disability. In summary, this is the first report that showed the wide variety of epileptic phenotypes in very rare patients with sequence variants in the *GABRE* gene.

We postulate that the *GABRE* gene is a likely candidate gene for epilepsies of different forms and severities. Our studies lay the grounds for a better understanding of novel molecular genetic factors related to these clinical features. They may also complement the current knowledge of pharmaco‐biochemical activities of GABA_A_ receptors and may be useful to improve current therapies or develop novel antiepileptic drug treatments in the future.

## 5 CONSENT FOR PUBLICATION

The consent for publication is available from the corresponding author on reasonable request.

## CONFLICT OF INTEREST

The authors declared that they have no conflict of interest.

## AUTHOR CONTRIBUTIONS

Fenja Markus: performed molecular research investigations and analyses (P1), mainly wrote the manuscript. Chloé Angelini: collected clinical data (P2), performed exome analyses (P2), and wrote parts of the manuscript. Aurelien Trimouille: shared clinical and genetic data (P2), performed clinical investigations and exome sequencing and data analyses (P2). Gabrielle Rudolf: performed molecular research investigations and analyses (P4). Gaetan Lesca: performed molecular research investigations and analyses (P4). Cyril Goizet: performed clinical investigations, shared clinical data (P2). Eulalie Lasseau: contributed to exome sequencing and analyses (P2). Benoit Arveiler: contributed to exome sequencing and analyses (P2). Marjon van Slegtenhorst: performed exome sequencing and data analyses (P3). Alice S. Brooks: performed clinical investigations (P3), shared clinical and genetic data (P3). Rami Abou Jamra: supported interpretations of clinical and genetic data. Georg‐Christoph Korenke: performed clinical investigations (P1), shared clinical data. John Neidhardt: shared clinical and genetic data (P1), contributions to the manuscript. Marta Owczarek‐Lipska: performed exome sequencing and analyses (P1), coordination of data exchange with cooperation partners and coordination of this study.

## Supporting information

Supplementary Material

## Data Availability

The data sets used and/or analyzed during this study are available from the corresponding author on reasonable request.
