# Supplementary material for: Rare variants in the GABAA receptor subunit ε identified in patients with a wide spectrum of epileptic phenotypes
Source: Mol Genet Genomic Med. 2020 Jun 25;8(9):e1388. doi: 10.1002/mgg3.1388 (PMC7507344; doi:10.1002/mgg3.1388)
Supplement: Supplementary file 1 — Supplementary Material [file MGG3-8-e1388-s001.docx]

**Supplementary data**


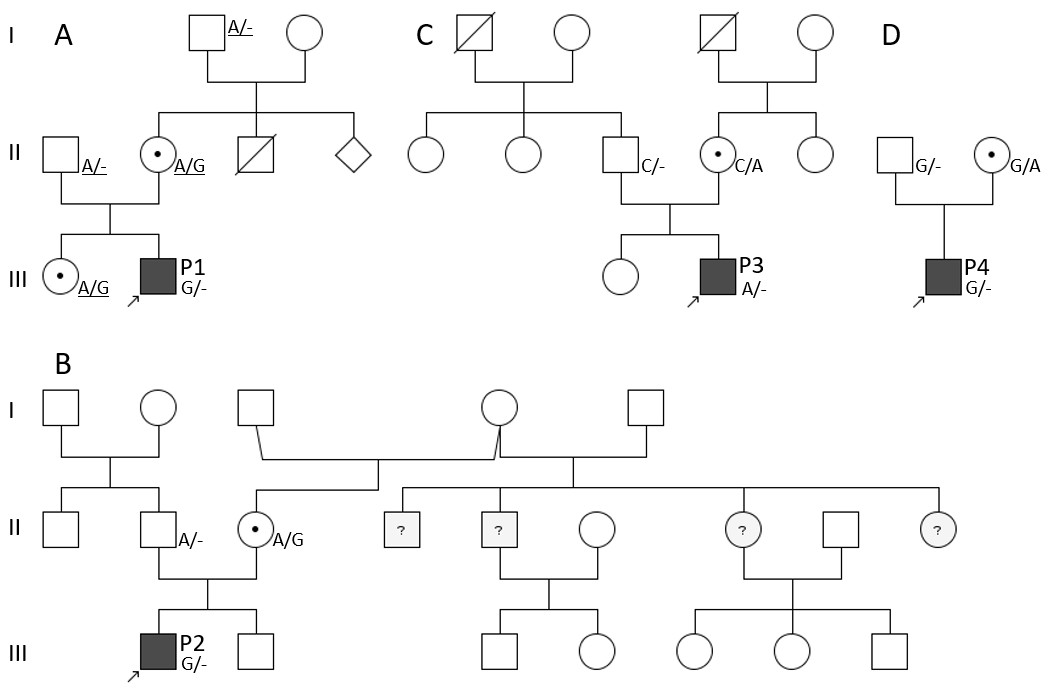


Figure S1 Extensive pedigree information of examined families.

The pedigrees show families of P1 (**A**), P2 (**B**), P3 (**C**) and P4 (**D**).

Symbols: I-III – generation 1-3, squares – males, circles – females, black solid squares with arrows – patients affected with epilepsy, rhombus – unknown gender, solid white icons – healthy individuals, icons with dot – known carrier, icons with question mark – mild intellectual deficiency, crossed out icons – deceased. The underlined genotypes were identified by Sanger sequencing and the remaining genotypes by (trio-)WES.

Table S1 Overview of sequence variants and *in-silico* protein prediction scores.

| Patients  *in-silico*  predictions | P1 | P2 | P3 | P4 |
| --- | --- | --- | --- | --- |
| *GABRE*-variant cDNA | c.664G>A | c.1A>G | c.399C>A | c.1015G>A |
| GABRE-variant protein | p.Glu222Lys | p.Met1? | p.Tyr133* | p.Val349Ile |
| Inheritance | X-linked | X-linked | X-linked | X-linked |
| GnomAD | 0.00004409 | 0.00001550 | 0.00001091 | NA |
| SIFT | deleterious | deleterious | NA | NA |
| M-CAP | possibly pathogenic | NA | NA | possibly pathogenic |
| Mutation Tester | disease causing | disease causing | disease causing | NA |
| MetaDome | intolerant | NA | NA | slightly intolerant |

NA – not accessible

Sequence variants were annotated at transcript NM_004961.3/NP_004952.2

**Table S2 General information about the sequencing variants.**

| **Data analyses** | | | **Patients** | | | |
| --- | --- | --- | --- | --- | --- | --- |
| **All variants** | **Filtering** | **Variants** | **P1^W^** | **P2^T^** | **P3 ^T^** | **P4 ^T^** |
| Variants w/out filtering |  | total amount | 58802 | 45193 | 44594 | 58251 |
|  |  | heterozygous | 34887 | 28213 | NA | 30018 |
|  |  | homozygous | 22886 | 16980 | NA | 12108 |
|  |  | hemizygous | 603 | 484 | NA | NA |
|  |  | de-novo | * | NA | NA | 60 |
| Variants with filtering | MAF < 1-1.5% | total amount | 2254 | 8010 | 273 | 12144 |
|  |  | heterozygous | 1747 | 5595 | 270 | 7916 |
|  |  | homozygous | 436 | 2415 | 0 | 1701 |
|  |  | hemizygous | 13 | 82 | 1 | NA |
|  |  | de-novo | * | NA | 2 | 22 |
|  | reads > 10 | total amount | 1216 | 7258 | NA | NA |
|  |  | heterozygous | 65 | 5281 | NA | NA |
|  |  | homozygous | 1100 | 1977 | NA | NA |
|  |  | hemizygous | 6 | 69 | NA | NA |
|  |  | de-novo | * | NA | NA | NA |
|  | High impact  (stop, splice) | total amount | 19 | 12 | 10 | 19 |
|  |  | heterozygous | 17 | 10 | 9 | 14 |
|  |  | homozygous | 2 | 0 | 0 | 5 |
|  |  | hemizygous | 0 | 2 | 1 | NA |
|  |  | de-novo | * | 0 | 0 | 13 |
|  | Moderate impact  (missense) | total amount | 282 | 25 | 167 | 46 |
|  |  | heterozygous | 260 | 22 | 167 | 42 |
|  |  | homozygous | 16 | 0 | 0 | 4 |
|  |  | hemizygous | 2 | 2 | 0 | NA |
|  |  | de-novo | * | 0 | 2 | 4 |
|  | Pathogenic | total amount | 1 | 1 | NA | 1 |
|  |  | heterozygous | 1 | 0 | NA | 1 |
|  |  | homozygous | 0 | 0 | NA | 0 |
|  |  | hemizygous | 0 | 1 | NA | 0 |
|  |  | de-novo | * | 0 | NA | 0 |

Abbreviations: W - single-WES analyses, T - trio-WES analyses, * - base on the single-WES data it was only possible to verify heterozygous variants. None of these variants was plausible candidate for further familiar co-segregation analyses with Sanger sequencing, NA – data not available.
